# Pathogenic role and clinical significance of neutrophils and neutrophil extracellular traps in idiopathic inflammatory myopathies

**DOI:** 10.1007/s10238-024-01384-2

**Published:** 2024-05-30

**Authors:** Ruiting Liu, Hongjiang Liu, Leiyi Yang, Changpei Li, Geng Yin, Qibing Xie

**Affiliations:** 1 Department of Rheumatology and Immunology, West China Hospital, Sichuan University, Chengdu, China; 2 Health Management Center, General Practice Medical Center, West China Hospital, Sichuan University, Chengdu, China

**Keywords:** Idiopathic inflammatory myopathies, Neutrophil extracellular traps, Low-density granulocytes, Autoimmune diseases

## Abstract

Idiopathic inflammatory myopathies (IIM) are a heterogeneous group of chronic autoimmune diseases characterized by muscle damage and extramuscular symptoms, including specific skin rash, arthritis, interstitial lung disease, and cardiac involvement. While the etiology and pathogenesis of IIM are not yet fully understood, emerging evidence suggests that neutrophils and neutrophil extracellular traps (NETs) have a role in the pathogenesis. Recent research has identified increased levels of circulating and tissue neutrophils as well as NETs in patients with IIM; these contribute to the activation of the type I and type II interferons pathway. During active IIM disease, myositis-specific antibodies are associated with the formation and incomplete degradation of NETs, leading to damage in the lungs, muscles, and blood vessels of patients. This review focuses on the pathogenic role and clinical significance of neutrophils and NETs in IIM, and it includes a discussion of potential targeted treatment strategies.

## Introduction

Idiopathic inflammatory myopathies (IIM) are a heterogeneous group of autoimmune diseases characterized by chronic inflammation of the muscles [1]. Common symptoms include muscle weakness, low muscle endurance, and myalgia as well as extramuscular manifestations, such as rash, arthritis, interstitial lung disease (ILD), and cardiac involvement [2]. These manifestations highlight the systemic inflammatory nature of IIM. The IIM group includes dermatomyositis (DM), immune-mediated necrotizing myopathy, inclusion body myositis, anti-synthase syndrome (ASS), polymyositis (PM), and overlap myositis [1, 3]. As a collective group, IIM is thought to be the result of an interaction between genetic and environmental risk factors in the relative absence of protective factors, but its exact etiology and pathogenesis remain unknown [4].

Although the study of neutrophil dysregulation and neutrophil extracellular traps (NETs) in IIM is still in its early stages, growing evidence suggests their significant role in the disease’s pathogenesis. In other systemic autoimmune diseases, including rheumatoid arthritis (RA), systemic lupus erythematosus (SLE), and anti-neutrophil cytoplasmic antibody-associated vasculitis (AAV), neutrophils enhance the ability to form NETs, advancing pathogenesis [5–7]. Patients with IIM exhibit increased neutrophils and NETs in both circulation and tissues. Several proteins associated with NETs, such as neutrophil elastase (NE), peptidylarginine deiminase (PAD), and antimicrobial peptide LL-37, indicate the presence of NETs in IIM [8]. Enhanced NET formation is particularly evident in the neutrophil subpopulation known as low-density granulocytes (LDGs) [5]. The activation of the interferon (IFN) pathway and promotion of cytokine production by NETs in IIM can induce both adaptive immune responses and autoimmune reactions [9, 10]. Furthermore, the presence of myositis-specific antibodies (MSAs) has been linked to excessive NET formation [11]. The dysregulation of NETs affects the lung, muscle, and blood vessels. Therefore, effective treatment for individual patients requires a deeper understanding of the molecular pathways underlying the pathogenesis and identification of relevant biomarkers. The objective of this review was to provide a summary of the pathogenic role of neutrophils and their derivatives in IIM and provide new insights into the treatment of patients with IIM.

## IIM and inflammation

Serologic features of IIM include high levels of serum autoantibodies and muscle enzymes, while histologic features include the infiltration of single nucleated cells and inflammatory cells in the skeletal muscle tissue [12, 13]. In IIM, the intramuscular vascular system actively participates in leukocyte recruitment, as evidenced by the changes in vascularity and the high expression of pro-inflammatory cytokines and chemokines in endothelial cells. Muscle cells produce pro-inflammatory cytokines and chemokines [14], and inflammation, when strictly regulated, is integral in muscle repair and regeneration. However, in the event that inflammation becomes chronic and dysregulated, it may exert enduring effects on muscle injury and regeneration [15, 16]. During chronic inflammation, persistent recruitment and activation of neutrophils, as well as impaired clearance of these cells, contribute to the exacerbation of inflammation, although they do not serve as the primary cause [17]. The activation and clearance of neutrophils serve as indicators that differentiate between stages of inflammation and regression in models of muscle injury and regeneration [18].

## Neutrophils and NETs

Neutrophils, the most abundant leukocytes, are a crucial aspect of host defense. They move through the circulatory system to sites of infection, where they recognize and eliminate invading pathogens [19]. However, neutrophils cause the release of proteases, reactive oxygen species (ROS), and other molecules that promote inflammation and tissue damage through inappropriate activation. Furthermore, activated neutrophils release, expose, or generate neoepitopes that have the potential to disrupt immune tolerance. This disruption may result in the production of autoantibodies, which are commonly observed in numerous autoimmune diseases [20].

In 2004, Brinkmann et al. introduced the concept of NETosis, which refers to the release of NETs by neutrophils [21]. NETs are lattice-like structures released into the extracellular space that are composed of nucleic acids, histones, and proteins derived from neutrophil particles [20]. They can be induced by a variety of factors, including pathogens, activated platelets, activated endothelial cells, complement proteins, autoantibodies, immune complexes, monosodium urate crystals, and multiple cytokines. Several pathways contributing to NET formation have been described in the literature, such as the generation of mitochondria-derived ROS, NADPH oxidase 2 (NOX2)-dependent production of superoxide, and the deamination of arginine residues in histones through peptidylarginine deiminase 4 (PAD4) [22, 23].

In the context of IIM, NET formation is associated with FcγR-mediated phagocytosis, C-type lectin receptor signaling, Toll-like receptor (TLR) signaling, and activation of the complement cascade [18, 24]. FcγRIIa (CD32a) and FcγRIIIb (CD16b) are consistently expressed by human neutrophils, and the activation of these receptors via interaction with immune complexes can induce the release of NETs [24]. Because NETs contain both nuclear and mitochondrial DNA, they influence inflammation when the latter is released into the extracellular space [22, 23]. The significance of NETs as a source of modified self-antigens and immune stimulatory molecules, along with their role in driving an enhanced type I interferon response, has been extensively emphasized by numerous studies [5–7, 25].

Damage-associated molecular patterns (DAMPs) initiate and enhance non-infectious inflammatory responses. A recent study proposed that NETs may be a new type of DAMP, because the most important NET components or substances closely related to NETs are DAMPs and are involved in the pathogenesis of IIM [8].

## Low-density granulocytes

As a pro-inflammatory subtype of neutrophils, LDGs have a distinct gene array profile compared to that of autologous normal density neutrophils. They are involved in the pathogenesis of various systemic autoimmune diseases, including SLE and AAV, and they spontaneously form NETs at a higher rate than that of autologous normal density neutrophils [5, 26]. These NETs synthesized by LDGs are more immunostimulatory and interfering [26, 27]. Moreover, LDGs have the capacity to overproduce type I IFNs, IFN-g, and tumor necrosis factor-α (TNF-*α*), which induce more neutrophils to generate NETs in vivo [5]. In the case of IIM, LDGs may cause injury in a similar manner to lupus LDGs, with increased levels of circulating LDGs and NETs, as well as enhanced NET formation [28].

## Neutrophils and NETs in myositis

### Lung injury

The diseases DM, ASS, and PM are types of IIM that frequently affect the lungs. ILD severely affects the prognosis of patients with IIM and is recognized as a major contributor to mortality [29]. The clinically amyopathic dermatomyositis (CADM) is characterized by a distinctive cutaneous rash and minimal or absent muscle inflammation, placing it within a distinct subset of DM [30]. One study reported that 60–80% of patients with CADM have ILD [31]. Many of these patients rapidly develop respiratory failure and die within 2–6 months of diagnosis [4]. ILD is also one of the most common clinical features of ASS. The prevalence of ILD in ASS is as high as 80%, and the incidence of rapidly progressive interstitial lung disease (RP-ILD) is at least that of DM [32]. Although the exact cause of myositis-associated ILD is unknown, neutrophils are considered a potential contributor to disease progression.

Several reports have shown that neutrophils in bronchoalveolar lavage fluid (BALF) are associated with a poor clinical course for patients with PM/DM [33, 34]. Elevated levels of lymphocytes and neutrophils were reported in DM patients with RP-ILD [35]. Furthermore, a significant increase in neutrophils in BALF was found in patients with DM-ILD with poor prognosis [33]. A captivating investigation assessed the levels of human neutrophil peptides (HNPs) *α*-defensins in patients with myositis-associated ILD, revealing that plasma and BALF HNPs may serve as indicators of the disease activity in myositis-associated ILD, offering an alternative perspective on the involvement of neutrophils in myositis pathogenesis [36]. The study conducted by Gono et al. revealed that PM/DM patients with ILD exhibited elevated serum levels of IL-4, IL-6, IL-8, IL-10, TNF-α, and CXCL10 prior to treatment in comparison with PM/DM patients without ILD [37]. Their study, as well as another, demonstrated that neutrophilic and M1-macrophage-driven cytokines, and M2-macrophage-driven cytokines, were connected with the pathogenesis of PM/DM-ILD [38]. In other research, NETs were found to be crucial in promoting neutrophil infiltration and causing lung injury [39, 40].

A study conducted in 2014 was the first to identify increased formation of NETs in DM/PM patients, as well as incomplete degradation of over-formed NETs due to reduced deoxyribonuclease I (DNase I) activity in ILD patients. This suggests that aberrant regulation of NETs is involved in the pathogenesis of DM/PM and may be one of the factors triggering and exacerbating ILD [11]. Also, NETs stimulate plasmacytoid dendritic cells to release type I IFN, which further disrupts the cytokine network [41, 42], leading to continuous NET formation. If these excess NETs are not cleared to halt the cycle, they can damage to the lung endothelium and disrupt homeostasis [43]. Additionally, NETs have the potential to accelerate the activation of lung fibroblasts and promote their differentiation into a myofibroblast phenotype in vitro [7]. In vivo, NETs were detected in lung biopsy specimens from ILD patients in close proximity to fibroblasts expressing *α*-smooth muscle actin (*α*-SMA) [3]. This suggests that the activation of lung fibroblasts by NETs contributes to the development of ILD. In addition, certain components of NETs, such as histones, may directly hurt vascular endothelial cells and epithelial cells [5, 44]. Lung tissue is vulnerable to attack by invading microorganisms and autoimmune responses, and the formation of NETs typically occurs in the airway lumen; therefore, dysregulated regulation of NETs could magnify the inflammatory response and induce an immune cascade in the lung tissue [45, 46]. Excessive formation of NETs has been implicated in myositis-associated ILD, although the underlying mechanisms remain unclear.

Numerous research studies have provided evidence of the role played by NETs in expediting the advancement of ILD within living organisms and their contribution to the development of pulmonary fibrosis (PF) [45, 47]. Then, ILD leads to the development of PF, which is characterized by lung fibroblast (LF) aggregation, proliferation, activation, and excessive deposition of extracellular matrix (ECM) proteins [48]. Myofibroblasts (MFs) are a specific subtype of LF distinguished by their expression of *α*-SMA and ECM proteins [49]. Lung fibroblasts transform into the MF phenotype. The activation, proliferation, and differentiation of LFs contribute to the progression of PF. Additionally, NETs and their components have a role in the activation of LFs and their differentiation into MFs. The TLR9-miR-7-Smad2 signaling pathway may be involved in this differentiation process [47]. Several studies have demonstrated that fibroblast activation and differentiation into the myofibroblast phenotype is induced by NETs treated with fibrosis-related drugs or generic NET inducers [47]. Collectively, this research highlights the involvement of NETs in the development and progression of PF.

### Muscle injury

Neutrophils are important in both muscle damage and the subsequent inflammatory response that is necessary for muscle repair. However, excessive activation of neutrophils exacerbates initial muscle damage and hinders successful repair [50]. In a research conducted using an animal model, Suzuki et al. observed the presence of NETs and the infiltration of neutrophils in muscle injury induced by intramuscular injection of monosodium urate [51]. The potential contribution of NETs to muscle hyperalgesia and aseptic muscle damage has also been evidenced [52].

Additionally, neutrophils have gained attention in the pathogenesis of chronic muscle injury. In a recent study by Nickie Seto et al., NETs and neutrophil gene expression were measured in biopsies of patients with IIM, and they showed increased levels of circulating LDGs and NETs, as well as an enhanced ability of LDGs to form NETs. The clinical manifestations of the disease and a type I–II IFN profile are associated with an increased expression of neutrophil-related genes in skeletal muscle biopsies from individuals with DM [53]. The transcriptome dataset of muscle tissue from various subtypes of IIM patients was recently analyzed, confirming the pivotal role of the IFN signaling pathway and neutrophil-mediated immunity in the underlying pathological processes [18]. Activation of the type I IFN pathway exhibited a positive correlation with disease activity in DM and PM, while type II IFN signatures were found to be upregulated in ASS [54]. NETs can promote the secretion of type I IFN, and in turn, IFN-*α* can stimulate the formation of NETs [41]. Neutrophils cause myofiber damage both in vitro and in vivo [55], and oxidants released by these cells result in severe cellular damage.

In IIM, NETs do not affect myoblast proliferation but reduce myotube activity in a citrullinated histone-dependent manner. Furthermore, citrullinated histone H4 is more capable of damaging skeletal myotubes than is native histone H4 [53].The limitations of this experiment, however, lie in the author’s sole assessment of muscle fiber size following exposure to NETs, without concurrent evaluation of their functional activity. Histone H4 binds and dissolves vascular smooth muscle cells and destabilizes atherosclerotic plaques, according to a recent report [56]. The impact of NETs on the vasculature also contributes to muscle tissue damage and disrupts regeneration [53]. Abnormal neutrophil responses and NET formation may be important in IIM-related muscle injury, but further research is required.

### Blood vessels injury

Vascular inflammation affects inflammatory activation, with neutrophils actively participating in this process [38]. It is worth noting that NETs are frequently observed in the vicinity of vascular structures, and their presence is associated with in vivo evidence of vasculopathy. In a previous study by Carmona-Rivera C et al., both LDGs and NETs were found to have a crucial vasculopathic role, and endothelial cell apoptosis following exposure to NETs was primarily triggered by the presence of matrix metalloproteinase-9 [57].

Juvenile dermatomyositis (JDM) is a rare, vascular type of DM. A negative correlation between the levels of NETs and LDGs and periungual nailfold capillary density serves as a sign of vasculopathy in JDM [53]. In a recent study, elevated levels of NETs and LDGs were found in IIM patients with signs of vasculopathy, including skin ulcers, calcium deposits, and abnormal nail fold capillaroscopy [9]. In addition, other studies have demonstrated a significant association between LDGs levels and vascular damage in autoimmune diseases like SLE, as well as with poor treatment responses in AAV [7, 58].

It has been suggested that adults with JDM in childhood may result in premature cardiovascular injury [59]. A recent study highlighted the presence of calcium crystal-mediated NET disease in children with JDM. Other crystals, such as cholesterol and monosodium urate monohydrate crystals, can also induce NETs [60, 61] and contribute to the development of atherosclerosis and gout, respectively. However, it has not been investigated whether children with JDM have elevated levels of NETs, and it is unclear whether calcium crystals activate neutrophils to release NETs. The same study also found elevated levels of calprotectin and peroxidase in JDM patients and correlated them with disease activity, suggesting that neutrophils are involved in the pathogenesis of JDM and serve as biomarkers for monitoring disease progression [22]. Elevated calprotectin levels have been associated with cardiovascular disease [62], and neutrophil activation is known to cause endothelial damage and subsequently contribute to the development of atherosclerosis [57]. Furthermore, JDM patients experience significant dyslipidemia early in the disease and develop atherosclerosis and increased intima-media thickness as they transition into adulthood [59]. Further studies are needed to investigate the involvement of neutrophils and NETs in the atherosclerotic process in JDM.

## NETs and MSA

The comprehension of IIM has been enhanced through the identification of autoantibodies linked to specific clinical phenotypes. In 2005, Sato et al. initially validated the presence of anti-melanoma differentiation-associated gene 5 (MDA5) autoantibodies in CADM patients from Japan [63]. Anti-MDA5 autoantibodies have now been suggested to be associated with RP-ILD, skin ulcers, and arthritis in patients with DM [64, 65]. Anti-Jo-1, an enzyme responsible for synthesizing anti-histidine tRNA, is frequently observed in adult IIM and holds significance in relation to the anti-synthetase syndrome [66]. Specific autoantibodies have been discovered to induce enhanced NET formation in other diseases, such as anti-neutrophil cytoplasmic antibodies in AAV [67], anti-ribonucleoprotein autoantibodies in SLE [27], and anti-citrullinated protein antibodies in RA [6]. Several studies have reported that anti-MDA5 and anti-TIF1 antibodies have similar roles in IIM and are associated with enhanced NET formation in both circulation and tissues [53, 68]. Patients with anti-Jo-1 antibodies have abnormally low DNase I activity [11]. Further investigation is needed to understand the relationship between MSA and NETs, and this research could lead to the development of new interventions for the treatment of IIM.

## NETs associated proteins

### NE

Neutrophil elastase, a serine protease, is primarily stored in the aspergillus granules of neutrophils [69]. When neutrophils are stimulated by invading pathogens, NE is released into the extracellular space and serves multiple functions, including processing inflammatory mediators and binding to oxidants [70]. The enzyme not only disrupts ligand proteins and breaks down extracellular matrix components, but also activates matrix metalloproteinase 9, which further enhances its disruptive effect on the vascular system and promotes neutrophil migration [71–75]. Neutrophil elastase has been found to promote PF by activating tumor growth factor-beta (TGF-*β*) and recruiting inflammatory cells to the lungs [76, 77]. Additionally, recent research has identified other mechanisms, such as NE entering fibroblasts and affecting their proliferative and contractile properties, leading to their differentiation into myofibroblasts and cytoplasmic protein degradation [78]. Furthermore, NE has been shown to play a role in myotonic dystrophy by influencing the survival, proliferation, and differentiation of myogenic cells [79]. Previous studies have reported elevated levels of NE in patients with IIM [80]. Wu et al. conducted a study where they proposed the utilization of a novel index known as the ENR (elastase-to-neutrophil ratio), which quantifies the average elastase level per polymorphonuclear (PMN) cell by comparing serum PMN elastase levels with serum neutrophil counts [70]. They found that patients with active IIM had higher levels of elastase and ENR compared to in those of patients in remission [70]. However, the precise role of elastase in assessing disease activity in IIM patients requires further investigation.

### Histones

Histones, including H2A, H2B, H3, and H4, are highly conserved proteins with a small size and positive charge. They play a crucial role in facilitating the compaction of DNA within the nucleus and constitute approximately 70% of proteins bound to NETs [81]. The release of free histones in NETs is facilitated by DNase derived from endogenous and pathogenic sources. DNA-free histones are recognized as crucial mediators in the pathogenesis of acute organ injury, tissue damage resulting from trauma, and sepsis [82]. It is noteworthy that the pro-inflammatory effect of histones has recently been substantiated, and elevated levels of extracellular histones have been identified in various autoimmune and inflammatory disorders [83]. Histones have the ability to function as bactericidal elements within NETs or induce neutrophils to produce NETs in a manner that is dependent on the dosage [84]. Extracellular penetration of histone H4 into the neutrophil membrane induces a calcium influx, leading to sustained elevation of intracellular calcium levels that trigger respiratory burst response, myeloperoxidase release, and IL-8 secretion. Histone H3 also exerts a comparable impact on the activation of neutrophils [85]. The functional significance of NETs and their constituents in inflammatory myopathies remains elusive, despite the widely recognized increased presence of NETs and histones under these conditions.

### Peptidylarginine deiminase (PAD)

Citrullination is a post-translational process in which arginine residues are converted to citrulline, which is mediated by PAD enzymes. This process leads to chromatin decondensation [86]. Peptidylarginine deiminase, particularly PAD4, is responsible for histone citrullination, resulting in the formation of citrullination histone H3 (CitH3), which has a crucial role in the formation of NETs [87]. Previous studies have found guanosine proteins in various types of inflammatory arthritis, suggesting that guanosine formation is associated with inflammation [86–88]. Evidence of citrullination was found in the muscle tissue of patients with IIM [89]. NETs attenuated myotube viability in a citrulline-histone-dependent manner in IIM patients, but not in control muscle tissue [89]. A recent study suggested that CitH3 serves as a serological biomarker to distinguish patients with DM from healthy individuals, as the authors found significantly lower levels of CitH3 in the serum of DM patients compared to those of controls [90]. In addition, PAD4 is associated with the development of fibrosis in mouse models as well as in patients with ILD [91]. In the mouse model, the absence of NETs resulting from PAD4 deficiency might exert a protective effect against changes in tissue cell composition during fibrosis, but the exact mechanism remains unknown. During the release of NETs, PAD4 is released into the extracellular space, allowing for post-translational modification of proteins other than histones, which could affect the phenotype of lung fibroblasts [92]. Abnormal PAD activity and protein citrullination have been closely linked to various autoimmune diseases such as rheumatoid arthritis and multiple sclerosis [89], but research on IIM is still limited.

### LL-37

LL-37, an antimicrobial peptide with broad and complex anti-inflammatory and immunomodulatory effects, has recently gained attention in research owing to its ability to promote the progression of autoimmune diseases [8]. It has been found in neutrophils, initially in cyanophilic granules and later in NETs [93]. LL-37, a crucial component of NETs, converts its own DNA into an activator of TLR9 in human plasmacytoid dendritic cells. This subsequently triggers the release of IFN-α and exacerbates immune dysregulation observed in psoriasis and SLE [94]. The expression of LL-37 is elevated in the muscle tissue, pripheral blood, and skin of IIM patients [9], indicating that neutrophils are the main source of LL-37. This suggests that LL-37 is extruded into NETosis and may contribute to the activation of the type I IFN pathway in IIM [9]. Prolonged exposure of the immune system to type I IFN may disrupt immune tolerance and eventually lead to autoimmune disease. However, certain biological properties of LL-37 and its exact role in IIM remain unclear. Further studies are necessary to fully understand the effects of LL-37 on immune system function and explore its potential therapeutic uses.

## Clinical implications and future directions

Although glucocorticoids are used as a first-line therapy for patients with IIM, only a small percentage of patients achieve complete disease remission after undergoing glucocorticoid therapy [95, 96]. Also, immunosuppressive therapy has a wide-ranging impact on the immune system and often leads to adverse effects. Moreover, chronic inflammatory processes usually result in irreversible tissue damage. Consequently, there is a strong need for biomarkers and new treatments [1].

**Fig.1:**
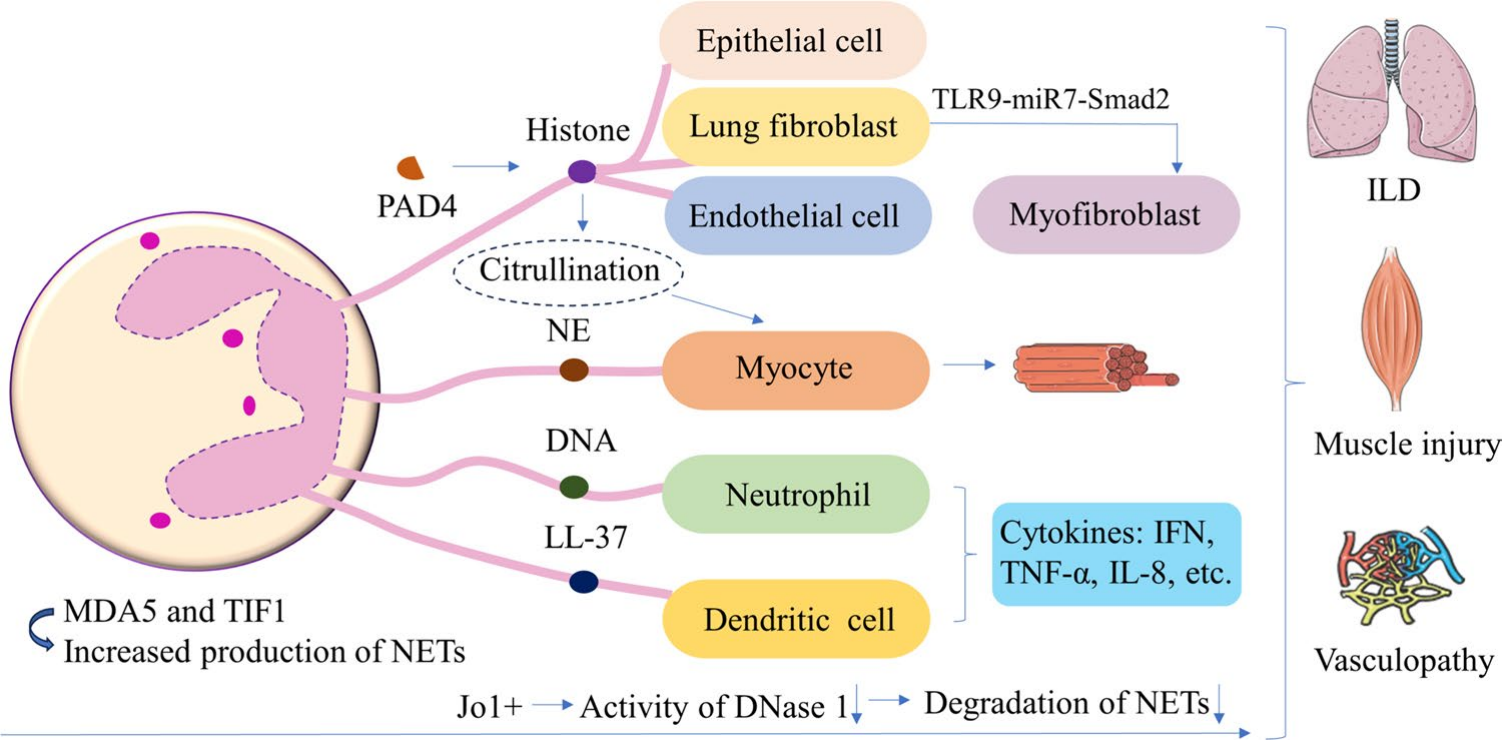
The pathogenic role of neutrophils and NETs in IIM

The ENR, neutrophil to lymphocyte ratio (NLR), and certain cytokines are associated with disease activity and could serve as useful biomarkers for assessing disease activity in patients with IIM [9, 70]. Additionally, a separate investigation revealed that patients exhibiting augmented neutrophil levels and elevated NLR in anti-TIF1g + DM demonstrate an increased susceptibility to cancer development [68]. As illustrated in Fig. 1, we have summarized the pathogenic role of neutrophils and NETs in myositis. Modulating neutrophil adhesion, inhibiting NET formation, and enhancing NET dissolution hold promise as innovative strategies for the treatment of IIM [50]. Previous studies have reported that polymyxin B-fixed fiber column hemoperfusion is effective in treating rapidly progressive interstitial pneumonia associated with CADM, possibly by promoting the uptake of circulating neutrophils [97]. In terms of pharmaceuticals, hydroxychloroquine has been found to inhibit TLR9 stimulation, thereby reducing NET release, and inhibitors of ROS production may indirectly decrease NET production [98]. The immunomodulatory effects of intravenous immunoglobulin therapy are exerted through intricate mechanisms, potentially involving the reduction in autoantibodies and inhibition of FcγR activation, which subsequently impacts the formation of NETs. Etanercept, known as a TNF inhibitor, can also exert therapeutic effects through its inhibitory action on FcγR [99]. Finally, strategies such as DNase I, antiproteases, anti-histone antibodies, and PAD inhibitors show promise in reducing NETs and could be explored for the treatment of IIM [47, 100].

## Conclusions

In conclusion, neutrophils and NETs have pivotal roles in the pathogenesis of IIM and hold promise as potential biomarkers and novel therapeutic targets. Further investigation into detailed molecular pathways and targeted therapies would offer additional options for the clinical treatment of IIM.
